# Estradiol suppresses tissue androgens and prostate cancer growth in castration resistant prostate cancer

**DOI:** 10.1186/1471-2407-10-244

**Published:** 2010-05-28

**Authors:** Bruce Montgomery, Peter S Nelson, Robert Vessella, Tom Kalhorn, David Hess, Eva Corey

**Affiliations:** 1 Department of Medicine, University of Washington School of Medicine, 1959 NE Pacific St. Seattle, WA, USA; 2 Department of Urology, University of Washington School of Medicine, 1959 NE Pacific St. Seattle, WA, USA; 3 Department of Medicinal Chemistry, University of Washington, 1959 NE Pacific St. Seattle, WA, USA; 4 Division of Human Biology and Clinical Research, Fred Hutchinson Cancer Research Center, 1100 Fairview Avenue North, Seattle, WA, USA; 5 Oregon National Primate Research Center, Oregon Health and Sciences University, 505 NW 185th Avenue, Beaverton, OR, USA

## Abstract

**Background:**

Estrogens suppress tumor growth in prostate cancer which progresses despite anorchid serum androgen levels, termed castration resistant prostate cancers (CRPC), although the mechanisms are unclear. We hypothesize that estrogen inhibits CRPC in anorchid animals by suppressing tumoral androgens, an effect independent of the estrogen receptor.

**Methods:**

The human CRPC xenograft LuCaP 35V was implanted into orchiectomized male SCID mice and established tumors were treated with placebo, 17β-estradiol or 17β-estradiol and estrogen receptor antagonist ICI 182,780. Effects of 17β-estradiol on tumor growth were evaluated and tissue testosterone (T) and dihydrotestosterone (DHT) evaluated by mass spectrometry.

**Results:**

Treatment of LuCaP 35V with 17β-estradiol slowed tumor growth compared to controls (tumor volume at day 21: 785 ± 81 mm^3 ^vs. 1195 ± 84 mm^3^, p = 0.002). Survival was also significantly improved in animals treated with 17β-estradiol (p = 0.03). The addition of the estrogen receptor antagonist ICI 182,780 did not significantly change survival or growth. 17β-estradiol in the presence and absence of ICI 182,780 suppressed tumor testosterone (T) and dihydrotestosterone (DHT) as assayed by mass spectrometry. Tissue androgens in placebo treated LuCaP 35V xenografts were; T = 0.71 ± 0.28 pg/mg and DHT = 1.73 ± 0.36 pg/mg. In 17β-estradiol treated LuCaP35V xenografts the tissue androgens were, T = 0.20 ± 0.10 pg/mg and DHT = 0.15 ± 0.15 pg/mg, (p < 0.001 vs. controls). Levels of T and DHT in control liver tissue were < 0.2 pg/mg.

**Conclusions:**

CRPC in anorchid animals maintains tumoral androgen levels despite castration. 17β-estradiol significantly suppressed tumor T and DHT and inhibits growth of CRPC in an estrogen receptor independent manner. The ability to manipulate tumoral androgens will be critical in the development and testing of agents targeting CRPC through tissue steroidogenesis.

## Background

The Nobel prize winning work of Huggins and Hodges described the use of estrogens and orchiectomy in the treatment of prostate cancer [1,2]. With the development of LHRH agonists, and the recognition that oral estrogens carried a significant risk of vascular complications, LHRH agonists supplanted estrogens as a primary treatment for advanced disease [3]. The use of diethylstilbestrol, and more recently transdermal estradiol, [4,5] remains relevant because estrogens can induce PSA response rates as high as 45% in selected patients with early "androgen-independent" or castration resistant prostate cancer (CRPC) [6]. Estrogens ameliorate toxicities associated with androgen deprivation by maintaining bone mineral density, suppressing hot flushes and improving both cognitive function and lipids in men with anorchid testosterone levels [7-9].

The relatively high response rate of castration resistant prostate cancer to secondary manipulations with estrogens and ketoconazole has been ascribed to suppression of adrenal androgen production [10,11]. Other investigators have suggested that estrogens directly inhibit growth of prostate cancer when administered in vitro in the absence of circulating hormones [12]. We have previously shown that 17β-estradiol suppressed CRPC growth and delayed mortality in multiple castration resistant xenograft models in vivo [13]. In these castrate animals, estrogen suppressed tumor growth despite the lack of circulating testosterone, dehydroepiandrosterone (DHEA) and androstenedione synthesis, a feature resulting from a lack of CYP17 in murine adrenal glands [14]. The estradiol inhibition of these human prostate cancer xenografts in castrated mice must therefore be independent of both testicular and adrenal androgens. Potential mechanisms of tumor suppression include receptor dependent inhibition through estrogen receptor β, [15,16]or receptor independent mechanisms such as induction of immune surveillance, or metabolism of 17β-estradiol to cytotoxic estrogens such as 2-methoxyestradiol [17]. We have demonstrated that metastases from men with progressive CRPC contain testosterone levels significantly higher than those in prostate cancer tissue from eugonadal men. Moreover, elevated tumoral androgens were associated with increased tumor transcripts encoding enzymes involved in the synthesis of androgens [18]. These studies suggested that prostate cancer can maintain intratumoral androgens to aid in tumor progression in CRPC. In the current study we hypothesize that 17β-estradiol might inhibit CRPC growth in anorchid hosts and suppress tumoral androgens by competitively inhibiting steroidogenesis from cholesterol, and reduce available tumoral androgens that drive growth [19-22]

In the current study we analyzed the effect of estradiol on tissue androgen levels in the castration resistant LuCaP 35V human prostate xenograft, demonstrating suppression of tumoral androgens. Tumor suppression in this model by estradiol was independent of the estrogen receptor, suggesting that competitive inhibition of androgen metabolism within tumor tissue is another potential mechanism by which estradiol and other estrogens inhibit prostate cancer growth.

## Methods

### Xenograft studies

All procedures were performed in compliance with the University of Washington Institutional Animal Care and Use Committee and NIH guidelines. Four- to 6-week-old male SCID mice (Fox Chase SCID mice, Charles River, Wilmington, MA) were used. The animals were orchiectomized at 8 weeks of age, and all animals were implanted with tumors at least 2 weeks after surgery. Tumor bits (20-30 mm^3^) of human castration resistant prostate tumor xenograft LuCaP 35V were implanted subcutaneously. LuCaP 35 originated from a lymph node prostate cancer metastasis from a patient with androgen resistant prostate cancer. Both the castration sensitive (CS) LuCaP 35 and isogenic, castration resistant LuCaP 35V express PSA and a wild-type androgen receptor [23]. Tumor growth was monitored by tumor measurements twice a week using calipers, and tumor volume was calculated as 0.5236 × LxHxW. When tumors reached 150 to 400 mm^3^, the animals were randomized into three groups. Group 1 (n = 10), the control group, were implanted with subcutaneous placebo pellets (Innovative Research of America, Sarasota, FL). Group 2 (n = 10): animals were supplemented with 17β-estradiol by subcutaneous implantation of slow-release Trocar pellets (90-day release, 0.36 mg; Innovative Research of America). Group 3 (n = 10): animals were implanted with 17β-estradiol pellets and the estrogen receptor antagonist ICI 182,780 was injected once a week (5 mg, subcutaneous injection) [24]. Control tissue for androgens was assayed from non-xenograft tissue from the same animals as well as from isogenic castration sensitive LuCaP 35 growing in eugonadal male mice. Effects of the treatments were monitored by biweekly measurements of tumor volume. Animals were sacrificed when tumors exceeded 1000 mm^3 ^or when the animals became compromised. Tumors were snap frozen in liquid nitrogen and stored at -80°C. Efficacy of the different treatment methods was assessed as average tumor volumes per group after 3 weeks of treatment. Growth delay was measured as the time from the start of treatment to the time for tumors to reach a volume of 1000 mm^3 ^[25]. All procedures were performed in compliance with the University of Washington Institutional Animal Care and Use Committee

### Statistical Analyses

The significance of differences in survival rate was tested using the log-rank statistic. Statistical significance between androgens in tissues of control and treated animals, differences in tumor volume at fixed intervals, and time to tumor volume of 1000 mm^3 ^were assayed using Student's t test; 95% confidence interval, and P ≤ 0.05 was considered significant.

### Steroid measurements

Androgen levels were determined by mass spectrometry (MS) using methods we have recently described [26]. In brief, frozen tissue samples were individually thawed, weighed, and homogenized in PBS. The homogenates were extracted with 8 ml of diethyl ether and the organic phase decanted after freezing the aqueous phase in a dry ice/ethanol bath. The organic phase was dried and concentrated with 2 × 0.5 ml ether washes under a stream of purified air. Each individual concentrated extract was dissolved in 1.0 ml redistilled ethanol and stored at -20°C until MS analysis. Samples were spiked with internal standards: 50 pg of deuterated (D3)-DHT and D3-testosterone, vortexed briefly, and evaporated to dryness. The residue was then reconstituted in 0.5 mL of water prior to extraction with methylene chloride. The organic phase was removed under nitrogen and the sample was dissolved in 0.1 M hydroxylamine hydrochloride in 50% MeOH/water, vortexed, and heated at 60° for 1 hour. Standards for DHT and testosterone were prepared in parallel. The resulting oximes were analyzed by LC-MS-MS using a Waters Aquity HPLC and Premier XE mass spectrometer (Milford, MA). Ions monitored were 350 > 309 and 347 > 306 for DHT-IS and DHT respectively, and 307 > 124 and 304 > 124 for testosterone-IS and testosterone respectively. This procedure resulted in a lower limit of quantitation of 100 and 500 attoMoles on column for testosterone and DHT respectively. Intra-assay coefficients of variation generated using human serum for high, mid and low-range samples were 3.5, 3.1 and 3.8% for testosterone and 6.3, 4.3 and 15.8% for DHT respectively. Data for tissue androgens was derived from 2-5 tumor samples per data point. Estradiol levels were performed on mouse serum collected at the time of sacrifice and assayed by ELISA (IBL, Hamburg).

## Results

### Effect of estradiol on tumor growth in orchiectomized mice

**Figure 1:**
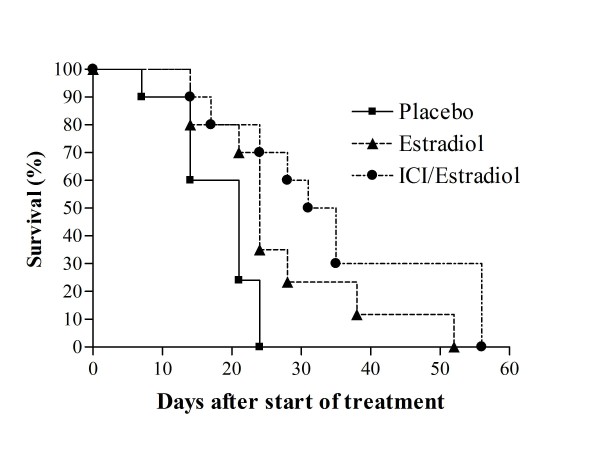
**Effect of 17β-estradiol or 17β-estradiol combined with ICI 182,780 on human CRPC xenograft growth in SCID mice**. Castrated SCID male mice were implanted with LuCaP 35V castration resistant cells and when tumors reached 150-400 mm^3^, treated with placebo pellets (n = 10): 17β-estradiol by subcutaneous pellets (n = 10); or 17β-estradiol pellets and ICI 182,780 (5 mg/kg SC once per week) as detailed in Materials and Methods. Data shown are Kaplan-Meier estimates of survival for tumor bearing animals treated with indicated agents.

Previous studies have shown that 17β-estradiol suppresses tumor growth in ovariectomized mice bearing prostate cancer xenografts LuCaP 35, LuCaP 49, LuCaP 58, LuCaP 73, and LNCaP [13]. These studies demonstrated that estradiol suppression of tumor growth was independent of effects on testicular androgens. We investigated whether estradiol suppression of castration resistant prostate cancer growth is dependent on estrogen receptor and independent of adrenal androgens. To address this question we examined the effect of 17β-estradiol on the castration resistant human prostate cancer xenograft LuCaP 35V in orchiectomized male mice in the presence and absence of the estrogen receptor antagonist ICI 182,780 (Faslodex). As previously discussed, adult male mice do not synthesize DHEA or androstenedione because the enzyme CYP17 is not expressed in the adult rodent adrenal gland [14]. Serum estradiol was collected from xenograft bearing animals at the time of sacrifice. Estradiol levels from control animals were 79.7 ± 67.9 pg/mL (mean ± standard deviation), levels from animals treated with 17β-estradiol alone were 636.9 ± 321.6, and animals treated with the combination of ICI 182,780 and 17β-Estradiol levels had had levels of 731.6 ± 508.5 pg/mL. Differences between the estradiol treated groups were not significant (p > 0.1). As shown on Fig. 1, 17β-estradiol prolongs survival of animals compared to controls (median survival 21 vs. 24 days, p = 0.03) and the administration of ICI 182,780 did not significantly change the effect of estradiol on survival (p = 0.14).

The mean tumor volumes at week 3 of treatment are shown in Table 1., The average tumor volume in the control group was significantly higher than in the 17β-estradiol treated group (Table 1, p = 0.02). The differences between 17β-estradiol and estradiol with ICI 182,780 were not statistically significant, though tumor volumes appeared slightly lower in animals treated with estradiol and ICI 182,780. Another way of quantitating effects on tumor growth is a comparison of the median time to reach a specified tumor volume [25]. This analysis shows that the time to a tumor volume of 1000 mm^3 ^(the prespecified point at which animals would be sacrificed) was also significantly different among the control group and the other two groups and reflects more rapid tumor growth (Table 1). Overall, 17β-estradiol extended the time to sacrifice by a median of 6 days (p = 0.002). The difference between the 17β-estradiol and estradiol/ICI 182,780 groups was not statistically different (p = 0.34). Although the use of ICI 182,780, did not result in differences in tumor growth which were statistically significant, there was a trend toward greater effect for the combination of estradiol with ICI 182,780. This might reflect additional efficacy of blocking estrogen receptor, or might reflect the higher serum estradiol levels in animals treated with estradiol and ICI 182,780. ICI 182,780 is metabolized via CYP3A4, which also metabolizes estradiol, suggesting one potential mechanism by which ICI 182,780 treatment might modulate estradiol levels The lack of a statistically significant difference in tumor growth with use of the estrogen receptor antagonist strongly suggests that tumor inhibition is independent of the estrogen receptor as well as androgens derived from testicular and adrenal sources.

**Table 1:** Tumor growth in response to 17β-estradiol and ICI 182,780

Groups	Tumor volume on Day 21 (mm3) (SE)	Days to 1000 mm3 (SE)
Control	1194.9 (83.9)	19.4 (1.2)
Estradiol	784.7 (81.1) *	25.7 (3.4) †,
Estradiol/ICI	675.2 (77.2) **	33.4 (4.4) ††

	* p = 0.002 vs. control	†, p = 0.05 vs. control
	** p = 0.001 vs. control.	†† p = 0.003 vs. control

### Estradiol effects on tumoral testosterone and DHT levels

**Figure 2:**
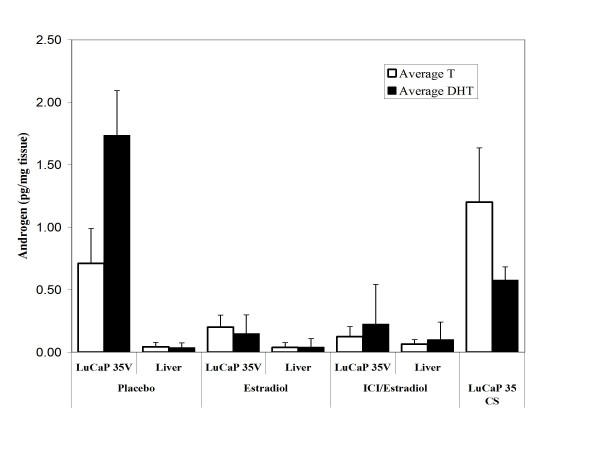
**Androgen levels in LuCaP prostate cancer human prostate cancer xenografts grown in castrate and intact SCID mice**. Testosterone and DHT levels were measured by mass spectrometry in castration sensitive (CS) and castration resistant variants of the LuCaP 35 xenograft. The castration resistant tumors were treated with agents as indicated (Fig. 1 and Materials and Methods). Androgen levels were also evaluated in liver tissue obtained from each set of treated castrate animals. Data represent three tissue samples per data point; *bars*, SE.

We next sought to determine how estradiol might be mediating an anti-tumor effect independent of circulating androgens, and independent of estrogen receptor. We have reported that metastatic prostate cancer from androgen deprived individuals contains supraphysiologic levels of tissue testosterone in the presence of increased transcripts encoding enzymes of steroidogenesis [18]. Estradiol can suppress the expression and enzyme activity of critical proteins in the steroidogenesis pathway. Sensitive components of the pathway include Steroidogenic acute regulatory protein (StAR), CYP17 and 3β-hydroxysteroid dehydrogenase [20-22]. To evaluate LuCaP 35V xenograft tissue androgens in the presence and absence of estradiol, we assayed tumoral testosterone and DHT by LC-MS/MS. Our results show that despite castration, the LuCaP 35V xenograft maintains tissue levels of androgens which approximate those found in tumor tissue in eugonadal men and are of a magnitude equivalent to those in the castration sensitive, isogenic line LuCaP 35 grown in eugonadal male mice. With estradiol exposure tumoral levels of androgens decline significantly (Fig. 2). Tissue testosterone levels in placebo treated LuCaP 35V xenografts were 0.71 ± 0.28 pg/mg and DHT concentrations were 1.73 ± 0.36 pg/mg. In estradiol treated LuCaPV35 the tissue testosterone concentrations were 0.20 ± 0.10 pg/mg and DHT levels were 0.15 ± 0.15 pg/mg, (p < 0.001 compared to controls). Levels in the tumors from animals treated with both estradiol and ICI 182,780 were not statistically different from those in tumors treated with estradiol alone. Liver from treated animals was used as control tissue as it lacks steroidogenic enzymes and does not contain elevated androgens in castrate animals [18]. Levels of T and DHT in control liver tissue from placebo and estradiol treated animals, were less than 0.2 pg/mg. Testosterone in castration sensitive tumors in intact mice was higher, and DHT was lower, than in the castrate animals. Although estradiol administration suppressed tissue androgens significantly, in association with a decrease in tumor growth, tumoral androgen levels did not reach the very low levels seen in control tissues. These residual androgens are still in the range expected to continue to activate the receptor, albeit significantly lower than those in tumors which were not treated with estradiol. These findings likely explain the incomplete tumor suppression seen with estradiol therapy. In summary, estradiol significantly suppresses both tumor growth and tumoral androgens in the absence of circulating testicular or adrenal androgens.

## Discussion

Estrogens have long played a role in the treatment of advanced prostate cancer, however the mechanism of efficacy in men who have already developed resistance to androgen-deprivation therapies has been uncertain. Estrogens induce feedback inhibition of LH secretion in patients with an intact hypothalamic-pituitary axis, resulting in decreased testosterone production. An early VA Cooperative Urology Group study compared the estrogen diethylstilbestrol (DES) to orchiectomy, and demonstrated that DES provided better cancer specific survival than orchiectomy, but cardiovascular mortality offset the improved disease control [27]. This study raised the possibility that estrogens might inhibit cancer growth independently of simple suppression of testicular androgen production. In support of that idea, our group has previously shown that estradiol inhibits multiple castration resistant human prostate cancer xenografts in castrated animals, however the mechanism remained ambiguous. This study was carried out to determine if estrogen inhibition of prostate cancer xenograft growth was estrogen receptor dependent, and whether estrogen might be mediating its effect by suppressing tissue androgens which could support tumor survival and proliferation. The data presented here strongly suggest that estradiol inhibition of tumor growth in this setting is estrogen receptor independent, as ICI 182,780 blocks both ERα and β signaling, yet did not abrogate estradiol mediated tumor suppression. In addition, estradiol clearly suppressed tumor androgen levels, an effect for which there is no plausible mechanism in castrated animals lacking functional adrenal CYP17. The xenografts used in these studies express wild type AR, not mutant AR and are therefore not expected to bind estradiol. It was first reported by Geller, and subsequently by others that prostate tissue in androgen deprived individuals maintains levels of DHT and testosterone which are between 20 and 50% those found in tissue from eugonadal men [28-32]. Maintenance of these tissue levels despite anorchid serum levels might occur either through sequestration of androgens, or by metabolism or synthesis of androgens from earlier precursors. The idea of tissue "intracrine" synthesis of androgen was proposed initially by Labrie et al [33]. This mechanism would incorporate the ability of estrogen to suppress tumor growth independent of gonadal or adrenal androgens. This report is the first to clearly demonstrate that prostate cancer xenografts maintain tissue androgens independent of endocrine function, in castrate hosts. It is also the first report that demonstrates that estradiol inhibition of tumor growth in this setting is not ER dependent and that estradiol effectively suppresses tumoral androgen levels, a result which might explain an ER independent mechanism of blocking castration resistant tumor growth. This data supports the concept that tissue androgens are not necessarily dependent on endocrine organ function. The importance of residual androgens is uncertain unless these tissue levels can be further suppressed to slow tumor growth, an effect which has been demonstrated in this study. Although not defined in these experiments, the most parsimonious explanation of the ability of estrogen to effect this change would be through its ability as a steroidal hormone to competitively inhibit the relatively promiscuous enzymes of steroidogenesis which might mediate tissue metabolism [19-22]. Multiple steroidal hormones are capable of interacting with steroid transporters and competitively inhibiting metabolism of other substrates, in any tissue which contains these enzymes.

Extrapolation of the results of xenograft studies to the biology of metastatic prostate cancer in man remains speculative. In patients with advanced prostate cancer, suppression of androgen production by orchiectomy or LHRH agonist administration has a transient, although significant impact on prostate cancer growth. Remarkably, despite the immediate clinical benefits of androgen deprivation, the tissue effects of suppressing serum androgen levels are much more modest. Our group has reported that benign prostate tissue as well as prostate cancers maintain physiologic levels of both testosterone and DHT despite effective suppression of serum androgens [31]. The ability of prostate tissue to maintain local hormone levels is reflected in the minimal change in the expression of androgen regulated gene expression in men with anorchid serum androgens [31]. These findings may explain the observation by Agus and colleagues that androgen deprivation in prostate cancer xenograft models demonstrated only transient cell cycle arrest, with little evidence of apoptosis, followed by relatively rapid progression [34]. The persistence of tissue androgens despite anorchid serum levels would be anticipated to continue to support prostate cancer cell survival, and growth over time.

Estrogens, including, ethinyl estradiol, DES, and transdermal estradiol remain an option for patients with both androgen dependent and castration resistant prostate cancer [4-6,35,36]. The current study provides evidence that a component of estrogen activity occurs though reductions of tissue androgen levels even when serum androgens are completely suppressed by castration. The effect is independent of estrogen receptor, and suggests that further investigation of how tumoral androgens play a role in progressive CRPC are warranted. Studies to elucidate the mechanism(s) by which tissue androgens are maintained will be critical for understanding the importance of this phenomenon. This becomes more important as agents become available which can more effectively modulate interactions between intracrine ligands and the AR, including high affinity AR antagonists and agents which interfere with enzymes of steroidogenesis [37,38]

## Conclusions

The current study demonstrates that estradiol slows tumor progression of CRPC xenografts grown in orchiectomized mice. The inhibition of tumor progression is associated with the ability of estradiol to inhibit intratumoral androgen levels, which at baseline are unexpectedly higher than would be expected in the context of no serum testosterone, and a lack of serum DHEA. The use of estradiol receptor antagonist did not block estradiol inhibition of tumor growth, nor did it change the suppression of tumor androgens, suggesting that both effects are independent of estrogen receptor. This study suggests that the known ability of secondary hormonal manipulations to suppress CRPC growth is related to inhibition of tumoral androgens, and in this specific study is independent of adrenal DHEA production. This has implications for the design of future therapies targeting different arms of steroidogenesis in prostate cancer as more effective treatment.

## Competing interests

The authors declare that they have no competing interests.

## Authors' contributions

BM conceived of the study, participated in its coordination, drafted the manuscript and performed the statistical analysis. PSN participated in data analysis and helped to draft the manuscript, TK performed the tissue androgen analysis, EC conceived of the study, participated in its coordination, data analysis and interpretation. All authors read and approved the final manuscript.

## Pre-publication history

The pre-publication history for this paper can be accessed here:

http://www.biomedcentral.com/1471-2407/10/244/prepub
